# Work–Life Enrichment and Interference Among Swedish Workers: Trends From 2016 Until the COVID-19 Pandemic

**DOI:** 10.3389/fpsyg.2022.854119

**Published:** 2022-07-13

**Authors:** Emma Brulin, Constanze Leineweber, Paraskevi Peristera

**Affiliations:** ^1^Department of Psychology, Stress Research Institute, Stockholm University, Stockholm, Sweden; ^2^Unit of Occupational Medicine, Institute of Environmental Medicine, Karolinska Institute, Solna, Sweden

**Keywords:** enrichment, interference, longitudinal data, pandemic, trends

## Abstract

The COVID-19 pandemic has altered workers' possibilities to combine work and private life. Work and private life could either interfere with each other, that is, when conflicting demands arise, or enrich, that is, when the two roles are beneficial to one another. Analyzing data from the Swedish Longitudinal Occupational Survey of Health through individual growth models, we investigated time trends of interference and enrichment between work and private life from 2016 through March to September 2020, which is during the first wave of the pandemic. The sample included workers who had remained in the same workplace throughout the study period and worked at least 30% of full time, reaching 5,465 individuals. In addition, we examined trends in level of interference and enrichment across gender and industries. Results showed that Life-to-work interference increased over time in the Swedish working population, but neither did work-to-life interference nor enrichment. We observed only marginal differences across gender. Also, in the industries of fine manufacturing and real-estate activities, a decrease in interference, work-to-life interference, and life-to-work interference, respectively, was observed. In the human health and social care industry, an increase in interference and life-to-work interference was seen. Our conclusion is that overall changes to the possibilities to balance work and private life have occurred for workers in Sweden during the first period of the pandemic. Further studies are needed to study development time trends throughout the pandemic and across different occupations.

## Introduction

With the spread of the SARS-CoV-2 during the COVID-19 pandemic, governments across the globe adopted different policies to minimize the spread of the virus, and many of those affected workers' possibilities to combine work with private life in all occupations (Howe et al., 2020). The Swedish governmental recommendation was that all employees who could work from home should do so. Lower-grade schools and pre-schools remained open, although with stricter restrictions on children and employees with symptoms. Upper-grade schools adapted to distance learning to varying degrees, with sudden transfers from “on place” to remote and “hybrid” teaching. As a result, workers were affected in various ways, partly depending on which industry they were employed in. For instance, workers in the health and childcare industries had to remain at the worksite but experienced longer work hours and higher work demands (Del Boca et al., 2020; Kaden, 2020; Liu et al., 2020; Morgantini et al., 2020). Employees in many industries, not at least in knowledge-intensive occupations, mainly were transferred to remote work. As a result of the transfer to remote work, employees in these industries often experience changes in their possibility of setting boundaries between work and private life (Howe et al., 2020; Sinclair et al., 2020; Vaziri et al., 2020). It is evident from previous research that workers' possibilities to set boundaries between work and private life has been affected by the pandemic; however, to what extent and in what direction is still unclear and needs further attention.

Overall research evidence from the pandemic is mainly restricted to cross-sectional data. It seems to focus either on employees working remotely during the pandemic or workers in the healthcare industry. In addition, previous evidence is based on countries that applied total lockdown during the pandemic, and therefore, it is unknown whether these findings apply to the Swedish context where no lockdown was proclaimed. In this study, we seek to explore if, and if so, in what way the COVID-19 pandemic has affected Swedish workers' possibilities to set boundaries between work and private life. We aim to explore trends over time in work–life interference and enrichment among the Swedish working population using longitudinal data from 2016 and throughout the start of the COVID-19 pandemic, that is, data for the SLOSH wave 2020 were collected between March/April to August/September of 2020. Moreover, as the pandemic has impacted industries and individual workers to a varying degree, we will examine trends by industry and gender.

### Interference, Enrichment, and Boundary Management

When one life role impedes and interferes with another life role, this is referred to as interference (Kossek and Lee, 2017). Interference works in two directions, work-to-private life interference (WLI) and private life-to-work interference (LWI). Previous work has shown that the intersection between work and private life does not only result in interference but also has synergistic and beneficial effects (Greenhaus and Powell, 2006; Allen and Martin, 2017). Positive spillover, enhancement, facilitation, and enrichment are concepts used to describe these gains between private life and work (Carlson et al., 2006, 2019; Williams et al., 2016). In this article, we study enrichment. As with interference, enrichment does work in two directions: work enriching private life (WLE) and private life-enriching working life (LWE) (Greenhaus and Powell, 2006).

The changing working conditions during the COVID-19 pandemic may have affected the way workers experience interference between work and private life and their possibility of setting boundaries between the two spheres. This supposition is in accordance with boundary theory, which proposes that individuals have various social, spatial, cognitive, emotional, and behavioral boundaries that they enact and uphold around their different life roles, for instance, work roles and home/family roles (Clark, 2000; Allen et al., 2014; Rothbard and Ollier-Malaterre, 2016). Boundaries can be either spatial, for example, the geographical distance between the office and the private outside office space, or emotional, for example, that emotions, positive or negative, spillover from one domain to the other (Rothbard and Ollier-Malaterre, 2016). The extent to which these boundaries exist depends on the individual's capacity or possibility for boundary management and preferences concerning keeping work and personal life separated (“segmentation”) or integrated (“integration”) (Clark, 2000; Mellner et al., 2015; Rothbard and Ollier-Malaterre, 2016). It may not always be possible for workers or in the worker's control to enact preferred boundaries (Mellner et al., 2015). Also, Clark (2000) argues that individuals' perceived possibility of contracting or expanding boundaries is more important than their capacity or personal traits. The COVID-19 pandemic could present both opportunities and constraints to workers' possibilities to exert boundary management.

When control over boundary management is lost, interference between work and private life can occur (Rothbard and Ollier-Malaterre, 2016). Societal changes, such as the COVID-19 pandemic, can increase permeability, making boundary management more critical. Boundary management segmentation (i.e., possibilities to keep work and private life separated) is associated with lower WLI and LWI but might contribute to higher WLE and LWE (Allen et al., 2014). However, evidence regarding changes in WLI during the pandemic is not consistent, with some studies indicating an increase in experienced WLI (Sinclair et al., 2020; Vaziri et al., 2020; Adisa et al., 2021; Craig and Churchill, 2021; Verweij et al., 2021) while others suggest the opposite (Schieman et al., 2021). The only study investigating LWI (Verweij et al., 2021) showed that LWI increased during lockdowns compared to at the start of the pandemic without lockdown. Evidence on enrichment during the pandemic is even more limited and comes mainly from qualitative studies outside Sweden. These studies show that the pandemic positively influenced employees' enrichment (Adisa et al., 2021; Verweij et al., 2021). In this study, we explore trends in interference and enrichment and in WLI, LWI, WLE, and LWE over the inspected time span. We propose the following research questions:

*Research question 1.1: What are the time trends of interference, WLI, LWI, enrichment, WLE, and LWE from 2016 to the beginning of the COVID-19 pandemic?*

*Research question 1.2: Are there any differences in time trends of interference, WLI, LWI, enrichment, WLE, and LWE in relation to the COVID-19 pandemic?*

### Time Trends in Interference and Enrichment in Different Industries

The impact of the pandemic in WLI and LWI for those remaining at the worksite and those working remotely is expected to differ. However, most evidence of teleworking compared to working on-site comes from before the pandemic. The rapid transition to social distancing in the early pandemic might have contributed to reduced control over boundaries to a varying degree across industries (Rothbard and Ollier-Malaterre, 2016; Allen et al., 2021).

Meanwhile, for workers who remained at their worksite during the pandemic, the long work hours and high workload they were, in many cases, exposed to might have increased psychological permeability, which could have impacted emotional and temporal boundary management (Byron, 2005; Grönlund, 2007; Fahlén, 2014). The sudden increase in work hours and demands might have contributed to the loss of boundary control. In addition, to the extreme workload during the pandemic, workers in the healthcare and education industry feared bringing the virus home (Mosheva et al., 2020), which could also have inflicted WLI.

Studies from before the pandemic show that remote work begets flexibility and autonomy and is suggested to be beneficial in reducing interference between work and private life (Hayman, 2009; Januszkiewicz, 2019). On the other side, studies from before the pandemic also show that remote work means that employees are in continuous connection to work through mobile phones or laptops, leading to more interference (Van der Lippe and Lippényi, 2020). Following boundary management, it is suggested that remote work is blurring boundaries between work and private life (Rothbard and Ollier-Malaterre, 2016). However, the evidence before the pandemic may not be valid since remote work was often seen as a privilege and a choice by employees and was mainly used to reduce conflicting demands between work and home. This has not been the case during the pandemic when remote work was mandatory and on a full-time basis (Anderson and Kelliher, 2020). Against the backdrop of the COVID-19 pandemic, the sudden enforced need to work from home while tending to children might have affected control over the boundary between work and private life (Allen et al., 2021). Hence, the transition to remote work or teleworking can adversely affect the individual worker's possibility of boundary management (Mellner et al., 2015).

In line with these suggestions, a Portuguese study showed that workers who were forced to work remotely, role overload, after-hours work-related technology use, and low job autonomy are related to increased levels of WLI (Andrade and Petiz Lousã, 2021). Furthermore, workers who were forced to work remotely following social distancing or lockdown often met new dimensions to remote work, for example, the need to help school children during distance learning or a partner sitting in the same room working (Anderson and Kelliher, 2020). These results may not apply to Sweden, where pre-schools and schools for younger pupils remain open.

So long, only two studies provided comparative analyses of the possibilities of combining work and private life for remote workers in relation to remaining workers. The first study showed that women who stayed at their worksite experienced more difficulties combining work and private life due to excessive workload (Del Boca et al., 2020). Using cross-country data from Europe, the second study showed that remote workers experienced more LWI than those who remained at their worksite, while no differences were found in relation to WLI (Blasko, 2020). To the best of our knowledge, no previous study has explored trends before and over the pandemic in relation to interference and enrichment in different industries. Hence, our second research question reads the following:

*Research question 2. Are there differences in how time trends in interference, WLI, LWI, enrichment, WLE, and LWE change in relation to the COVID-19 pandemic within different industries?*

### Trends in Interference and Enrichment Across Genders

Gender is an essential aspect of individuals' possibility to combine work with private life (Acker, 1990). In gender theory, it is proposed that men and women do gender and that gender is re-socialized in what men and women do (West and Zimmerman, 1987; Connell, 2002). This means that men and women are bound to act according to socially shaped ideas about what feminine or masculine is, for example, the notion that women are better at taking care of the housework and that men should provide for their families (Connell, 2002). A gender theoretical perspective is therefore essential in relation to interference and enrichment. With respect to both gender theory and boundary management, femininity and masculinity are also reflected in how men and women set boundaries between work and private life. As Shockley et al. (2017) argued, men and women tend to create stronger boundaries around the domain that typically affirm their gender. Strong boundaries around private life mediate the relationship between gender and WLI (Shockley et al., 2017).

Pre-pandemic studies on differences by gender in work and private life interference and enrichment are somewhat inconsistent. Some studies show that men report higher levels of interference (Fahlén, 2014; Lunau et al., 2014), while others show that women report more interference (McGinnity and Calvert, 2009; Lunau et al., 2014). Meanwhile, others conclude that there are no gender differences (Geurts and Demerouti, 2003). The mixed evidence can be due to differences in culture and gender expectations (Strandh and Nordenmark, 2006; Fahlén, 2014), differences in female labor market participation (Lunau et al., 2014; Hagqvist et al., 2017a), socioeconomic status, working hours, and level of education (Leineweber et al., 2013; Hagqvist, 2016). Studies from Sweden indicate that working women report slightly more interference than working men, especially when considering working hours (Leineweber et al., 2013).

Studies from other countries than Sweden indicate that the COVID-19 pandemic has impacted women's working life to a more considerable degree than men's working life (Cannito and Scavarda, 2020; Collins et al., 2021; Graham et al., 2021). Closed pre-school activities and distance teaching strategies in many countries inflicted foremost on mothers' possibilities to even remain working. This is indicated by studies showing that women reduced their work hours substantially during the pandemic (Collins et al., 2021; Craig and Churchill, 2021). Moreover, women working from home had to do home-schooling and take responsibility for the emotional welfare of children and keep children at home with the lightest symptom of illness (Anderson and Kelliher, 2020; Wenham et al., 2020). Women have also experienced an increased domestic workload during the pandemic (Adisa et al., 2021; Craig and Churchill, 2021; Hjálmsdóttir and Bjarnadóttir, 2021). Meanwhile, studies from the Netherlands and the United States show that fathers have taken on greater childcare and housework responsibility during the pandemic (Carlson et al., 2021; Yerkes et al., 2022). An Australian study showed that men who worked from home (as compared to women working from home) more often had a separate workspace and were less often disturbed during work hours (Graham et al., 2021). Italian women working from home experienced fewer boundaries between work and private life than their husbands (Cannito and Scavarda, 2020). Meanwhile, in a study by Yerkes et al. (2022), it is indicated that women in more gender-equal countries, with high female labor market participation, compared to women in less gender-equal countries, to a greater extent, struggled with combining work and private life during the pandemic. Van der Lippe and Lippényi (2020) establish that boundary management has shown to be more challenging to achieve for women in comparison to men in general and gender differences are more pronounced when men and women work from home. A European study indicates that female workers who remained at their worksite experienced more WLI than women with remote work during the pandemic. There was no difference between men who remained at their worksite or worked from home (Blasko, 2020). Thus, the previous studies from various countries indicate that COVID-19 has disrupted women's possibilities to set boundaries around work and private life to a greater extent than among men (Cannito and Scavarda, 2020; Collins et al., 2021; Graham et al., 2021). The fact that women have more difficulty achieving boundary management (Van der Lippe and Lippényi, 2020) makes us assume that the boundaries between the two domains are more often blurred among women, which causes additive strain. Therefore, exploring the additive effects between the two directions for men and women would be valuable.

Regarding enrichment, Beham et al. (2020) show that before the pandemic, women report higher levels of WLE. Hagqvist et al. (2021), on the contrary, found no difference in the level of WLE between men and women but a higher level of LWE among women in the time before the pandemic. During the pandemic, many women experienced positive aspects of working from home, allowing them to create a closer relationship with family (Adisa et al., 2021; Hjálmsdóttir and Bjarnadóttir, 2021), which would suggest more enrichment.

As far as we know, no similar studies have been conducted about men's experiences of interference and enrichment during the pandemic in Sweden. Evidence during the pandemic comes mostly from countries with more substantial restrictions than Sweden.

In this study, gender constructs are essential in two aspects. First, we know that the Swedish labor market is highly gender-segregated, with female workers more often found in industries, such as healthcare and childcare (Cerdas et al., 2019). That is, women work to a more considerable degree in industries where workers during the pandemic had to remain at work and that was marked by unprecedented demands. Second, although Sweden is considered a gender-equal country, work tasks, especially housework and childcare, are still gendered, and women tend to do the lion's share of housework (Hagqvist, 2016; Hagqvist et al., 2017b). Meanwhile, men only spend marginal more hours on paid work (Hagqvist et al., 2017b). Women who have the main responsibility for the home often have stronger boundaries around family life (Shockley et al., 2017). Hence, our third research question reads the following:

*Research question 3: Are there gender differences in how time trends in interference, WLI, LWI, enrichment, WLE, and LWE change in relation to the COVID-19 pandemic?*

## Methods

### Data

Data were drawn from the 2016, 2018, and 2020 waves of the Swedish Longitudinal Occupational Survey of Health (SLOSH). SLOSH is an approximately representative sample of the Swedish working population and includes questions about work organization, work environment, labor market participation, and health. Since 2006, data have been collected every second year by means of a postal questionnaire in two versions: one for those in paid work and one for those having left work or working <30% (for more details, see Magnusson Hanson et al., 2018). Data collection takes place from March/April to August/September each year. Thus, in 2020, the data collection covered the period when the pandemic started and, therefore, can capture related changes in working life. The sample for this study is restricted to those who answered the questionnaire for the working population in all three waves (n_2016_ = 13,572, n_2018_ = 11,553, and n_2020_ = 10,294) and who have been working at the same workplace at all entry points, reaching 5,465 individuals.

Ethics approval for the SLOSH data collection was obtained from the Regional Research Ethics Board in Stockholm (DNR: 2012/373-32/5, 2015/2187-32, 2017/2535-32, 2019-06331) and for this study from the Swedish Ethical Review Authority (DNR: 2019-00972).

### Measurement

Interference and enrichment were measured by a questionnaire originally developed by Fisher et al. (2009) and adapted to Swedish. Four statements measure WLI, while LWI, WLE, and LWE are in each case measured by three statements. Each statement was responded to on a five-point Likert scale reaching from “not at all” to “almost always.”

Although both interference and enrichment have support for bidirectional paths, some authors argue that the two directions result in an additive strain and should be measured as one concept (Crompton and Lyonette, 2006; Fahlén, 2014). As one study (Verweij et al., 2021) indicates that both directions of interference increased during the pandemic (no studies found on enrichment), it would be valuable to explore both the bi-direction and the additive strain. Therefore, in this study, interference and enrichment will be studied at two levels: first, direction by direction (WLI, LWI, WLE, and LWE, respectively), and second, concept by concept, that is, interference (WLI and LWI in combination) and enrichment (WLE and LWE in combination). Scales were constructed using the mean across items. A higher number indicates higher levels of interference and enrichment, respectively. Reliability tests showed high internal consistency for the respective construct (interference and enrichment) and direction (WLI, LWI, WLE, and LWE) for each wave varying between 0.75 and 0.91 (Table 1).

**Table 1:** Mean values, standard deviations (in parenthesis), and Cronbach's alpha for interference, WLI, LWI, enrichment, WLE, and LWE.

	**2016**		**2018**		**2020**	
	**Mean (St. D.)**	**Cronbach's alpha**	**Mean (St. D.)**	**Cronbach alpha**	**Mean (St. D.)**	**Cronbach alpha**
Interference	2.04 (0.62)	0.81	2.04 (0.60)	0.81	1.93 (0.61)	0.82
WLI	2.63 (0.97)	0.91	2.61 (0.99)	0.91	2.47 (0.96)	0.91
LWI	1.46 (0.59)	0.71	1.48 (0.59)	0.75	1.40 (0.55)	0.81
Enrichment	2.89 (0.72)	0.81	2.90 (0.71)	0.82	2.96 (0.74)	0.82
WLE	2.46 (0.88)	0.83	2.47 (0.87)	0.76	2.55 (0.89)	0.83
LWE	3.32 (0.85)	0.82	3.33 (0.84)	0.76	3.37 (0.86)	0.82

Control variables include gender (men and women), children living in the home (yes and no), having a partner (yes and no), work hours (hours/week), and age at the end of 2016. In addition, analyses were stratified based on gender and industry. Gender, age, and industry were derived through linkage to the longitudinal integrated database for health insurance and labor market studies (LISA). All other control variables were derived from the questionnaire. The variable industry is based on the Swedish Standard Industrial Classification (SNI) 2007 and regards the industry a person worked in during 2016. The SNI is based on NACE Rev.2 and classifies enterprises and workplaces according to the activity carried out. The SNI codes classify individuals according to five digits. Respondents were divided into 17 different industries by the first two digits in the SNI codes. A list of industries and the number of respondents in each industry are found in Supplementary Material.

### Statistical Analysis

First, the mean and SD for constructs (interference/enrichment) and directions (WLI, LWI, WLE, and LWE) for each wave were produced.

Next, individual growth curve models (IGC) were used to study developments over time for directions and constructs. Individual growth curve models allow for modeling within-person systematic change and between-person differences in developmental outcomes across different measurement waves over time and to capture both linear and non-linear change. As Shek and Ma (2011) described, we tested a series of models to decide on the model that best fit our data. First, an unconditional mean model was carried out (Model 1). After that, a linear growth curve model examined any significant variation in individual trends over time (Model 2). Next, we tested the quadratic rate of change by adding quadratic parameters for the time in Model 2 (Model 3). In Model 3, the quadratic term was not allowed to vary over time since we have only three-time points. We included time-variant and invariant covariates for the model with the best fit (Model 4). All models were estimated through maximum likelihood (ML). To decide about the best model in terms of fit, we calculated the critical value for the chi-square distribution for *p* < 0.05 using the log-likelihood (−2 log-likelihood: −2LL) and degrees of freedom (DF) for Models 1–3 following Field (2013). Finally, we run Model 4 separately for men and women and industries. Analyses were performed using the mixed model procedure in IBM SPSS 25.0.

## Results

The sample consisted of 42% men and 58% women. The mean age for the sample was 50.8 in 2016. About 80% of the respondents had a partner, and the number was the same throughout the study period. In 2016, 50% had children living at home. This share gradually reduced over the studied period to 44% in 2018 and 40% in 2020. Respondents worked broadly the same number of hours in 2016 and 2018. However, in 2020, work hours were reduced, and a larger share of the respondent worked <40 h.

### Observed Mean Patterns

**Figure 1:**
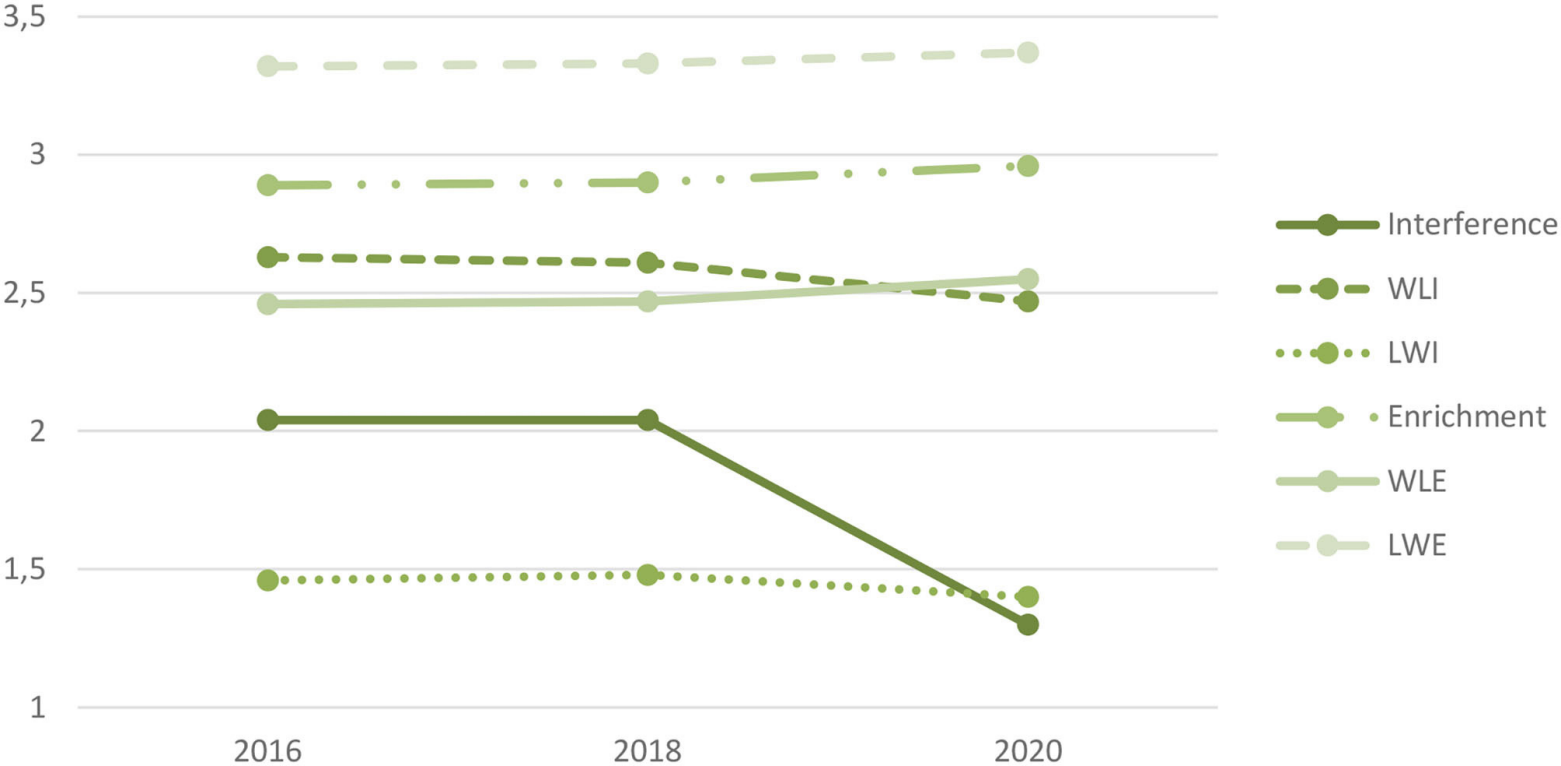
Mean values for interference, WLI, LWI, enrichment, WLE, and LWE for the studied period.

In Table 1, mean values and SDs (in parentheses), as well as internal consistency for the respective directions (WLI, LWI, WLE, and LWE) and constructs (interference and enrichment) for each year, are shown. Mean values for interference, WLI, LWI, enrichment, WLE, and LWE for each year are shown in Figure 1. Mean values for WLI and LWI are observed to decrease between 2016 and 2020. Looking at the mean values of interference, no change was observed between 2016 and 2018, while a decrease was seen in 2020. Mean values for WLE and LWE increased across the studied period. For enrichment, we observe increased mean values across the studied period.

### Individual Changes Over Time

In relation to research questions 1.1 and 1.2, the model fit statistics for the unconditional mean model (Model 1), the unconditional linear growth curve model (Model 2), and the unconditional quadratic growth curve model (Model 3) are shown in Table 2. The unconditional quadratic curve growth model (Model 3) was significantly better compared to the unconditional linear growth curve model for all constructs and directions. Therefore, we use the unconditional quadratic growth model as our basic growth model for all outcomes. We extend these models by including time-invariant covariates (Model 4, conditional growth model).

**Table 2:** Model fit with −2 log-likelihood for all models and respective outcome variables.

	**Model 1^**a**^**	**Model 2^**b**^**	**Model 3^**c**^**	**Model 4^**d**^**	**Change M1 and M2**	**Change M2 and M3**
Interference	26,063.9	25,764.0	25,670.4	24,558.1	299.9	93.61
WLI	41,083.4	40,795.8	40,745.3	39,086.0	287.6	50.52
LWI	25,814.7	25,710.3	25,644.9	24,385.3	104.41	65.41
Enrichment	32,414.3	32,305.5	32,289.3	31,268.7	108.76	16.23
WLE	39,432.4	39,310.9	39,296.7	38,130.0	121.52	14.21
LWE	39,173.1	39,126.8	39,122.6	37,826.7	46.33	4.11
DF^e^	3	6	7	22		
Critical value for *P* = 0.05					7.81	3.84

a *Unconditional mean model*.

b *Unconditional linear growth curve model*.

c *Unconditional Quadratic linear growth curve model*.

d *Conditional Quadratic linear growth curve model (model adjusted for covariates)*.

e *Degrees of freedom*.

Next, we present the estimated parameters from Model 4, that is, the conditional growth model that includes covariates (Table 3). As shown in Table 3, the first part describes the estimates of fixed effects, then the estimated parameters for the covariates, and finally the estimates of covariance parameters. The growth parameters, that is, the estimates of time and time squared, were found to be statistically significant only for LWI, while time was significant for interference. More specifically, we found a positive effect of the linear growth term (β_time = 0.34; SE = 0.12; *p* = 0.006), suggesting that LWI increased over time. A negative effect on the quadratic growth (β_time^2^ = −0.15; SE = 0.06; *p* = 0.010) was found, indicating that the rate of growth is changing less rapidly over time, producing lesser curvature in the represented trend. According to these results, LWI followed a curvilinear pattern. The significant linear growth in interference (β_time = 0.21; SE = 0.11; *p* = 0.049) indicated an increase over time.

**Table 3:** Quadratic growth curve for interference, WLI, LWI, enrichment, WLE, and LWE, respectively, including covariance variables (gender, having children, having partner, work hours, and age).

	**Interference**		**WLI**		**LWI**		**Enrichment**		**WLE**		**LWE**	
	**Est**	**Std. E**	**Est**	**Std. E**	**Est**	**Std E**	**Est**	**Std. E**	**Est**	**Std. E**	**Est**	**Std. E**
Intercept	2.08^*^	0.06	2.13^*^	0.10	2.02^*^	0.06	2.69^*^	0.07	2.10^*^	0.10	3.27^*^	0.06
Time	0.21^*^	0.11	0.07	0.18	0.34^*^	0.12	−0.01	0.12	0.11	0.17	−0.11	0.19
Time^2^	−0.10	0.05	−0.04	0.08	−0.15^*^	0.06	0.02	0.06	−0.02	0.08	0.03	0.09
Women (Ref. Men)	0.06^*^	0.02	0.23^*^	0.03	−0.12^*^	0.02	0.05^*^	0.02	0.04	0.02	0.06^*^	0.02
Children (Ref. No children)	0.08^*^	0.01	0.05^*^	0.02	0.13^*^	0.02	−0.01	0.02	0.01	0.02	−0.03	0.02
Partner (Ref. No Partner)	−0.05^*^	0.02	−0.03	0.03	−0.08^*^	0.02	0.10^*^	0.02	−0.01	0.03	0.22^*^	0.03
Workhours	0.08^*^	0.01	0.19^*^	0.01	−0.02^*^	0.01	0.01	0.01	0.00	0.01	0.02	0.02
Age	−0.01^*^	0.00	−0.00	0.00	−0.01^*^	0.00	0.00	0.00	0.01^*^	0.00	−0.00^*^	0.00
Women*Time	0.02	0.03	0.04	0.04	−0.00	0.03	−0.06	0.03	−0.07	0.04	−0.04	0.04
Women*Time^2^	−0.00	0.01	−0.02	0.02	0.00	0.01	0.03^*^	0.02	0.03	0.02	0.03	0.02
Children*Time	−0.04	0.03	−0.03	0.04	−0.05	0.03	−0.00	0.04	0.03	0.04	−0.03	0.05
Children*Time^2^	0.00	0.01	0.00	0.02	0.01	0.02	−0.00	0.02	−0.00	0.02	0.01	0.02
Partner*Time	0.00	0.03	−0.01	0.05	0.03	0.03	0.06	0.04	0.06	0.05	0.07	0.05
Partner*Time^2^	0.00	0.02	−0.00	0.02	0.00	0.02	−0.03	0.02	−0.02	0.02	−0.03	0.02
Workhours*Time	0.00	0.02	0.04	0.03	−0.03	0.02	−0.02	0.02	−0.06^*^	0.03	0.01	0.03
Workhours* Time^2^	−0.00	0.01	−0.02	0.01	0.02	0.01	0.01	0.01	0.03	0.01	−0.01	0.01
Age*Time	−0.00^*^	0.00	−0.00	0.00	−0.00^*^	0.00	0.00	0.00	−0.00	0.00	0.00	0.00
Age* Time^2^	0.00	0.00	0.00	0.00	0.00	0.00	−0.00	0.00	0.00^*^	0.00	0.00	0.00
Residual	0.12^*^	0.00	0.29^*^	0.01	0.15^*^	0.00	0.20^*^	0.00	0.30^*^	0.01	0.32^*^	0.01
Variance for intercept	0.26^*^	0.01	0.63^*^	0.02	0.19^*^	0.01	0.31^*^	0.01	0.46^*^	0.01	0.38^*^	0.01
Covariance for intercept and slope	−0.02^*^	0.00	−0.03^*^	0.01	−0.02^*^	0.00	−0.01^*^	0.00	−0.02^*^	0.01	−0.02^*^	0.01
Variance for slopes	0.01^*^	0.00	0.03^*^	0.00	0.01^*^	0.00	0.02^*^	0.00	0.03^*^	0.01	0.02^*^	0.00
−2LL	245,588.10		39,085.99		24,385.28		31,268.65		38,130.04		37,826.67	

* *p < 0.05*.

Finally, all the covariance parameters, that is, the random parameters associated with the intercept and slopes, shown in Table 3, were found to be statistically significant, suggesting that the variability in these parameters could be explained by between-individual predictors.

Several covariates significantly predicted interference and enrichment (constructs and directions) at baseline. More specifically, we found that women experienced more interference (β = 0.06; SE = 0.02; *p* < 0.001) and WLI (β = 0.23; SE = 0.03; *p* < 0.001) and less LWI (β = −0.12; SE = 0.02; *p* < 0.001) in relation to men. Furthermore, at baseline, women experienced marginally more enrichment (β = 0.05; SE = 0.02; *p* = 0.013) and LWE (β = 0.06; SE = 0.02; *p* = 0.016), while no sex differences were found for WLE. Having children living at home significantly increased interference (β = 0.08; SE = 0.01; *p* < 0.001), WIL (β = 0.05; SE = 0.02; *p* = 0.046), and LIW (β = 0.13; SE = 0.02; *p* < 0.001), but not enrichment (constructs and directions). Having a partner was protective against interference (β = −0.05; SE = 0.02; *p* = 0.003) and LWI (β = −0.08; SE = 0.02; *p* < 0.001) and increased enrichment (β = 0.10; SE = 0.02; *p* < 0.001) and LWE (β = 0.22; SE = 0.03; *p* < 0.001). A higher number of weekly hours worked are related to more interference (β = 0.08; SE = 0.01; *p* < 0.001) and WLI (β = 0.19; SE = 0.01; *p* < 0.001), but also to lower levels of LWI (β = −0.02; SE = 0.01; *p* = 0.021). Number of hours worked per week had no significant effect on enrichment (construct and direction).

In the case of linear and quadratic changes, we found that gender predicted the quadratic change in enrichment and work hours predicted the linear change in WLE. Age was also found to be associated with some of the outcomes, however, with very low estimates.

### Differences Across Industry

In this section, we will answer research question 2, Table 4 shows that the industry of other service activities showed significant result for a positive linear growth term for interference (β_time = −1.20; SE = 0.55; *p* = 0.032) and negative quadratic growth (β_time = 0.56; SE = 0.27; *p* = 0.039). The real-estate activity industry was presented with a significant negative linear term for interference (β_time = −1.92; SE = 0.89; *p* = 0.031) and for LWI (β_time = −1.90; SE = 0.98; *p* = 0.043), but the quadratic term was presented as non-significant for both outcome variables. The industry of fine manufacturing showed significant results for a negative linear growth term for WLI (β_time = −2.96; SE = 1.43; *p* = 0.041) but non-significant quadratic growth.

**Table 4:** Quadratic growth curve per industry^a^.

**Outcome variable**	**Industry**	**N**	**Time^**b**^**	**Time^**2b**^**
Interference^a^	Other service activity	485	−1.20 (0.55)^*^	0.56 (0.27)^*^
	Real-estate activity	401	−1.92 (0.89)^*^	0.81 (0.43)
	Human health and social work activities	3,461	0.69 (0.24)^*^	−0.32 (0.11)^*^
WLI^a^	Fine manufacturing	181	−2.96 (1.43)^*^	1.31 (0.70)
	Art, entertainment, and recreation	235	2.99 (1.50)^*^	−1.54 (0.74)^*^
LWI^a^	Information and communication	669	1.94 (0.65)^*^	−0.81 (0.31)^*^
	Human health and social work activities	3,460	0.95 (0.24)^*^	−0.44 (0.12)^*^
	Real-estate activity	401	−1.84 (0.90)^*^	0.66 (0.44)

a *Only industries with significant results for time and/or time^2^ are shown*.

b *Models adjusted for gender, having children, having partner, work hours, and age*.

* *p < 0.05*.

For the industries of human health and social work activities, arts, entertainment, recreation, and information and communication, the estimates show a reversed situation. The linear growth terms were significant and positive, and the quadratic growth terms were significant and negative, meaning that in these industries, the respective measurements increased over time, but the rate of change slowed down over time. The industry of human health and social work activities is presented with significant results for interference (β_time = 0.69; SE = 0.24; *p* = 0.004 and β_time^2^ = −0.32; SE = 0.11; *p* = 0.006) and for LWI (β_time = 0.95; SE = 0.24; *p* < 0.001 and β_time^2^ = −0.44; SE = 0.12; *p* < 0.000). Art, entertainment, and recreation are presented with significant result for WLI (β_time = 3.14; SE = 1.61; *p* = 0.049 and β_time^2^ = −1.56; SE = 0.80; *p* = 0.041). The information and communication industry are presented with significant result for LWI (β_time = 1.94; SE = 0.61; *p* = 0.002 and β_time^2^ = −0.81; SE = 0.29; *p* = 0.006).

### Differences Across Genders

To answer research question 3, gender-separate analyses were carried out for Model 4. These analyses showed only small differences between men and women in development over time (Table 5). For men, LWI showed a significant positive change in the linear term (β_time = 0.49; SE = 0.17; *p* = 0.005) and a negative quadratic rate of change (β_time^2^ = −0.23; SE = 0.06; *p* = 0.006). For women, we found a positive effect of the linear growth term (β_time = 0.31; SE = 0.14; *p* = 0.026) and a negative effect on the quadratic growth (β_time^2^ = −0.14; SE = 0.07; *p* = 0.042) for interference. In both cases, a deceleration in the rate of change occurred.

**Table 5:** Quadratic growth curve for interference, WLI, LWI, enrichment, WLE, and LWE for men and women separately.

		**Interference** ^**a**^		**WLI** ^**a**^		**LWI** ^**a**^		**Enrichment** ^**a**^		**WLE** ^**a**^		**LWE** ^**a**^	
		**Est**	**Std. E**	**Est**	**Std. E**	**Est**	**Std E**	**Est**	**Std. E**	**Est**	**Std. E**	**Est**	**Std. E**
Men	Time	0.10	0.16	−0.30	0.23	0.49*	0.17	0.03	0.21	0.26	0.24	−0.22	0.26
	Time^2^	−0.04	0.08	0.14	0.11	−0.23*	0.08	0.01	0.10	−0.06	0.12	0.08	0.13
Women	Time	0.31*	0.14	0.38	0.22	0.23	0.15	−0.09	0.17	−0.08	0.22	−0.09	0.22
	Time^2^	−0.14*	0.07	−0.19	0.10	−0.09	0.07	0.05	0.08	0.09	0.10	0.06	0.11

a Models adjusted for having children, having partner, work hours, and age. ^*^p < 0.05.

## Discussion

In this study, we explored trends over time in work–life interference and enrichment both in constructs (interference and enrichment) and in directions (WLI, LWI, WLE, and LWE) among the Swedish working population using longitudinal data from 2016 and throughout the start of the COVID-19 pandemic, that is, data were collected during spring and summer of 2020. In response to the study aim, we proposed three research questions.

In relation to our first research question (1.1 and 1.2), our findings suggest that Swedish workers have experienced a curvilinear pattern in LWI over time and a linear increase in interference over time, but no change in, WLI, enrichment, WLE, or LWE was found. These results show that LWI first increases in 2016 and then decreases in 2020, indicating a reduction in LWI during the initial phase of the COVID-19 pandemic. Interference, on the contrary, increased steadily over time. Our results both support and contradict results from studies conducted in countries in which workers have experienced decreased possibilities to combine work and private life during the pandemic (Sinclair et al., 2020; Adisa et al., 2021; Craig and Churchill, 2021; Hjálmsdóttir and Bjarnadóttir, 2021; Schieman et al., 2021; Verweij et al., 2021). The Swedish recommendation for social distancing that endorsed schools and childcare to keep open seems to have positively affected workers' experience of LWI, at least in the initial phase of the pandemic. This could change when more parents had to stay at home to care for children with colds or symptoms throughout the pandemic (schools were closed to a larger degree). For instance, The Swedish Social Insurance Agency (2021) reports that in parts of 2020 and 2021, parents have used care of children's allowances more than before. The fact that interference showed significant linear growth supports this idea, but more research is needed. Over time, the continued development throughout the pandemic in the experienced level of interference and potential health effect thereof should be further explored in cross-country and longitudinal studies.

In response to our second research question, some variations were found within some of the studied industries but not all. The industry of other service activities had a u-shaped developmental trend over time, meaning that levels decreased. This is an industry where, at large, workers have been able to continue the work as usual. However, for the industries of human health and social work activities, arts, entertainment, recreation, and information and communication, the results suggest that the level of interference, WLI, and LWI, respectively, first increased over time and then decreased in relation to the pandemic. These are industries where workers have had a significant increase in workload or experienced other dramatical cuts in their work life. Despite the increased work demands and longer work hours that workers in the industry of human health and social work activities have experienced during the pandemic (Liu et al., 2020; Morgantini et al., 2020), this study showed decreased levels of interference and LWI. This study also shows that the industry of information and communication has experienced less LWI. As this study was conducted at the beginning of the pandemic, changes might not have had an effect yet. Another scenario could be that those with the most increased workload during the first wave of the pandemic might not have participated in the study. The art, entertainment, and recreation industry have been highly impacted by pandemic regulations as people have been bound to their homes and not been able to enjoy public events. As such, workers in this industry in Sweden have experienced a decrease in WLI. The trends over time within industries did not show a coherent result. One reason for this could be that the last time point was early in the pandemic, that is, data were collected between March/April and August/September of 2020. Another could be that there are large variations within industries that motivate within and across occupation variations. Moreover, in this study, we did not include preference in boundary management. Allen et al. (2021) explored the relationship between segmentation preferences in boundary management and the balance between work and private life among those who worked remotely due to the pandemic. Contradicting the authors' hypothesis, those who preferred high segmentation experienced more balance between the two domains when forced to work remotely. Our results strongly encourage more studies across and within industries to gain more knowledge on the various ways the COVID-19 pandemic has affected workers' possibilities for boundary management.

The third research question focused on gender differences. The results show marginal differences between men and women. While interference increased for women (but neither LWI nor WLI), LWI increased for the male working population in the last time point during the first period of the pandemic. In relation to the minor differences found across men and women, there could be many reasons for this (Shockley et al., 2017). First, as mentioned above, the Swedish labor market is gender-segregated. Men and women are found in different industries (Cerdas et al., 2019) and therefore can have been exposed to remote work and remain at work to different degrees. Men and women can either benefit or not benefit from the changes in each industry to various degrees, which in turn impact their possibilities for boundary management (Shockley et al., 2017). To further explore whether this is true, future studies need to study gender differences across industries and the eventual impact the COVID-19 pandemic had on changes in the organization of work and private life for men and women in the respective industries. Also, research should further explore whether this could have had implications on interference and enrichment for men and women during the COVID-19 pandemic.

Second, the socially constructed gender norms in the organization of work and private life have been emphasized during the pandemic, which has been proposed in the previous studies (Cannito and Scavarda, 2020; Collins et al., 2021; Craig and Churchill, 2021; Graham et al., 2021). Stronger bonds around the family are associated with lower WLI (Shockley et al., 2017). However, when permeability between work and private life ceases to exist or is extremely strained, as during the pandemic, it is plausible that it contributed to an additive strain between WLI and LWI for women. This is indicated in the significant increase in interference for women compared to men. This additive effect and potential gender differences should be further explored in future studies. In comparison with many other countries, in Sweden, schools and childcare facilities were kept open during the pandemic. This meant that parents could remain working instead of leaving work to care for or home school children, which has been seen in other countries (Anderson and Kelliher, 2020; Adisa et al., 2021; Hjálmsdóttir and Bjarnadóttir, 2021; Verweij et al., 2021). More studies should be conducted on parental responsibilities in Sweden during the pandemic, and the impact childcare and distance learning have on parents' possibilities to combine work and private life.

Contrary to the previous studies, which showed that individuals also perceived a positive spillover between work and private life during the pandemic (Adisa et al., 2021; Hjálmsdóttir and Bjarnadóttir, 2021; Verweij et al., 2021), our study showed no such pattern for enrichment, LWE, nor WLE. In those previous studies, the positive aspect mentioned was more time with family. However, since school and childcare remained open in Sweden, parents might not have experienced that positive aspect.

This study makes essential contributions to existing knowledge in several aspects. Literature concerning changes in work and private life over time and during the pandemic is still scarce, and since strategies to reduce the impact of the SARS-CoV-2 differed substantially across countries, it is important to conduct specific-country analysis to capture the effect of the pandemic on work and private life. Furthermore, this study has measured interference and enrichment at three different levels, enabling us to have a more detailed picture of the dimensions of the work–family interaction that was changed over time. Lastly, this study is based on longitudinal data, enabling us to explore patterns of change and the dynamics of individual behavior. However, more studies that include more measure points after the first wave of the COVID-19 pandemic would be needed to draw firmer conclusions.

### Strengths and Limitations

The major strengths of this study are the longitudinal design and the fact that the last entry point was conducted during the first phase of the pandemic. The SLOSH cohort is large, which implies robust results. We also use a robust statistical method that overcomes the limitations of traditional repeated measures techniques (e.g., repeated measure ANOVA). This study, however, comes also with some limitations. As surveys in general, SLOSH is answered by somewhat more women, those who are older, persons born in Sweden, married, and who have a university education, and therefore, the generalizability of our results may be limited. Second, the measurements of interference and enrichment come from the general SLOSH survey, which does not particularly address questions about changes due to the pandemic. However, this could also be regarded as a strength, as the measurement is not influenced by direct questions about the influence of the pandemic. Third, this study captures only the short-term impact of the pandemic. Since many answered the survey rather at the beginning of the pandemic (during early spring 2020), the effects might not have become quite so visible yet. Meanwhile, Collins et al. (2021) show that employees' work time significantly changed between February and April of 2020. More studies with later measurements and specifically addressed questions about the changes due to the pandemic are needed to better understand the effects of the COVID-19 pandemic. Fourth, although we have the possibility to group respondents in industries according to occupational registers, these groups are not homogenous and include occupations that were more or less affected by proclaims of social distancing. Also, it might be that our results underestimate the effects of the pandemic for some occupations, as those most affected by it (extremely increased workload in certain healthcare occupations) might not have answered the questionnaire. Nevertheless, no previous studies have explored variations across industries and the potential differences across workers within them.

## Conclusion

This study found curvilinear trends in LWI over time, which implies that Swedish workers during the first wave of the pandemic experienced reduced demands from spillover from private life to work. However, some variations were observed within different industries, indicating that boundary management has been affected differently for workers from various occupations. In future research, occupations should be acknowledged when conducting research on interference and enrichment during the pandemic. Lastly, only marginal gender differences were observed. Overall, this study suggests that social distancing, in contrast to lockdown, seems to have reduced adverse effects on interference for workers, which should be considered in future pandemic plans.

## Data Availability Statement

Information on and eventual access to the Swedish Longitudinal Occupational Survey of Health can be found at: http://www.idear-net.net/slosh/in-english. Requests to access these datasets should be directed to http://www.idear-net.net/slosh/in-english.

## Author Contributions

EB contributed to the design of the paper, analysis, and writing. CL and PP assisted in developing the manuscript design and in the analytical process and provided valuable comments on the manuscript. All authors contributed to the article and approved the submitted version.

## Funding

This data collection was funded by the Swedish Research Council (# 2015-06013) and the REWHARD consortium supported by the Swedish Research Council (# 2017-00624). This study was funded by the Swedish Research Council for Health, Working Life, and Welfare (# 2018-01190 and # 2021-00215).

## Conflict of Interest

The authors declare that the research was conducted in the absence of any commercial or financial relationships that could be construed as a potential conflict of interest.

## Publisher's Note

All claims expressed in this article are solely those of the authors and do not necessarily represent those of their affiliated organizations, or those of the publisher, the editors and the reviewers. Any product that may be evaluated in this article, or claim that may be made by its manufacturer, is not guaranteed or endorsed by the publisher.
