# Supplementary material for: Work–Life Enrichment and Interference Among Swedish Workers: Trends From 2016 Until the COVID-19 Pandemic
Source: Front Psychol. 2022 Jul 13;13:854119. doi: 10.3389/fpsyg.2022.854119 (PMC9328076; doi:10.3389/fpsyg.2022.854119)
Supplement: Supplementary file 1 [file Table_1.pdf]

Supplementary material

Table 1. Number of respondents in each industry

	N	Per cent (%)
Agriculture, forestry and fishing	68	1,2
Mining and quarrying and manufacturing	635	11,1
Fine manufacturing	62	1,1
Electricity, gas, and water supply	70	1,2
Construction	250	4,4
Wholesale and retail of motor vehicles and motorcycles	425	7,4
Transport and storage	225	3,9
Accommodation and food service activities	94	1,6
Information and communication	227	4,0
Financial and insurance activities	129	2,3
Real estate activities	140	2,4
Professional, scientific and technical activities	372	6,5
Administrative and support service activities	652	11,4
Public administration and defence compulsory social security	165	2,9
Education	918	16,1
Human health and social work activities	1195	20,9
Arts, entertainment, and recreation	82	1,4
